# Flourishing and Self-Control in Adolescence: The Role of Perceived Parenting

**DOI:** 10.3390/ijerph20166568

**Published:** 2023-08-12

**Authors:** Maria Mirandi, Adriana Lis, Claudia Mazzeschi, Jian-Bin Li, Luciana Pagano Salmi, Elisa Delvecchio

**Affiliations:** 1 Department of Philosophy, Social Sciences and Education, University of Perugia, 06123 Perugia, Italylucianapsalmi@gmail.com (L.P.S.); 2 Department of Developmental Psychology and Socialization, University of Padova, 35131 Padua, Italy; 3 Department of Early Childhood Education, The Education University of Hong Kong, New Territories, Hong Kong

**Keywords:** self-control, flourishing, perceived maternal and paternal parenting, adolescence

## Abstract

Self-control is the ability to control thoughts, emotions, and impulses to pursuit of long-term goals. Adolescents with high self-control experience higher flourishing levels. The latter refers to the fulfillment of needs for competence, relationship, and self-acceptance, as well as the possession of psychological capital such as flow and commitment. High levels of self-control also seem to be linked to a positive relationship with parents, which is crucial in adolescent flourishing. However, few studies investigated the association between flourishing, self-control, and perceived parenting in adolescence. The aim of this study was to investigate how the ability to exert self-control and the need to perceive and preserve a responsive relationship with parents would facilitate adolescents’ experience of higher flourishing levels. The relationships among self-control, adolescents’ perception of some paternal and maternal dimensions (closeness, communication, and parents’ peer approval), and flourishing were analyzed in a sample of 335 Italian adolescents. Self-control was positively correlated with flourishing and the adolescent’s perception of maternal and paternal dimensions. The PROCESS model showed that perceived maternal and paternal dimensions mediate the relationship between self-control and flourishing. These findings show the importance of self-control and parenting dimensions in promoting flourishing during adolescence.

## 1. Introduction

Adolescence is a specific period of life characterized by significant changes at all levels of functioning, with profound and lasting implications on flourishing [1], self-control [2,3], and relationships with parents [4,5]. Flourishing was conceptualized by Diener and colleagues [6] as the fulfillment of the needs for competence, relationship, and self-acceptance, as well as the possession of psychological capital such as flow and commitment. Self-control, instead, is defined as the ability to control thoughts, emotions, and impulses to follow social norms and personal values and support the pursuit of long-term goals [7,8,9,10]. Adolescents with high self-control skills were supposed to establish better relationships with their parents because they are able to compromise, express emotions, and be more socially adaptive, constructive, reflective, and compliant [11]. Literature [12,13,14] suggests that self-control and interpersonal relationships promote adolescents’ flourishing [15,16,17]. However, there are few empirical studies on flourishing in adolescents in the literature [12,13], and they never provided an operational definition of flourishing. The current study aims to fill this gap in a group of Italian adolescents.

### 1.1. Flourishing

The term flourishing refers to the good life, feeling good, and high effectiveness of functioning in human actions [18]. Flourishing is considered central to human development and well-being. Positive ethics for public health aim to promote the functioning of strong public health systems for resilient individuals and communities. From this point of view, flourishing, rooted in equal respect for all human beings and encompassing human capabilities, is a shared moral goal and a common good for all people, and is therefore to be considered a problem of public health [19]. The term flourishing was originally born within two approaches of well-being: the hedonic (HWB, subjective well-being—SWB—the subjective experience of happiness and life satisfaction) and the eudaimonic (EWB, psychological well-being, PWB: psychological functioning, good relationships with others, and self-actualization) [20,21,22,23]. Theoretical debates about the hedonic and eudemonic approaches included conceptualizing flourishing in different ways [24,25]. Some authors captured only the eudaimonic component, others defined flourishing as reflecting both hedonic and eudaimonic components of subjective well-being [26,27]. This prompted several theorists to use the concept of flourishing [6] interchangeably with the concept of ‘psychological well-being’ [20,21], which is based instead on the idea of universal human needs and optimal functioning. Currently, there exist four primary theoretical approaches to flourishing. This study specifically adopts the reference approach proposed by Diener et al. [6], which summarizes how, in order to flourish in life, one must function well in both intrapersonal and interpersonal aspects, and flourishing is conceptualized as possessing psychological capital such as flow and engagement. Some studies stressed the importance of flourishing in adolescents [28,29,30], but never used empirical measures of flourishing, understood as the fulfillment of needs for competence, relationship, and self-acceptance [6]. Aspects of flourishing in adolescence include personal attitudes or beliefs, positive interpersonal relationships, and task-related characteristics such as diligence and initiative [31]. These aspects influence the achievement of central goals in adolescence, such as personal growth and increased autonomy [32]. Failure to achieve flourishing in adolescence may affect some related areas, such as attachment relationships, curiosity, and interest in learning. In addition, flourishing as a measure of achievement may operate as a possible mediator between family adversity and health problems in adulthood [31]. Diener et al. [6] developed a specific measure of this construct, the Flourishing Scale. However, despite governments increasingly recognizing the importance of measuring this construct [28], this scale is infrequently used for measuring flourishing in adolescence.

### 1.2. Self-Control

Self-control is considered one of the main strengths of human beings, as it consists of a set of skills that enable one to regulate thoughts, behaviors, emotions, and impulses and to act on the basis of one’s own personal and interpersonal goals in order to support the pursuit of long-term goals [7,8,9,10,12]. Adequate self-control in early life is associated with a vast range of positive long-term outcomes, including good physical and mental health, higher education levels, and better career opportunities [2]. In turn, low self-control is linked to problems in school or work functioning, experiences of psychological distress, and the development of mental health problems [3]. Self-control steadily improves from childhood to adulthood, but this skill is still maturing during adolescence [33]. At this stage of life, self-control is essential because it increases the level of positivity by controlling thoughts, emotions, and behavior [12]. Adolescents with high self-control skills feel more comfortable asking others for help because they have fewer concerns about giving up their personal control. They are better able to relate to others, compromise, and express emotions, making them more adaptable, constructive, reflective, and compliant. Therefore, adolescents with high self-control are happier and healthier than those with low self-control [34,35,36,37], which is associated with poor relational success [38,39]. Based on what was suggested in the literature, it would be compelling to investigate the relationship between self-control and flourishing with specific measures; especially because most studies investigated this relationship by considering flourishing as a measure of positive emotions or happiness, and not as a separate construct as suggested by Diener et al. [6].

### 1.3. Perceived Maternal and Paternal Parenting

Most studies in the literature examined parenting from the parents’ perspective, partly ignoring adolescents’ perceptions and emphasizing maternal parenting over father parenting [40]. Mothers are attributed a central role in their children’s development since they spend more time with them, are more involved in their lives, and bear the majority of the burden for duties, such as daily care [6]. It is important to investigate perceived parenting in adolescence since in this phase adolescents face numerous challenges to acquire greater autonomy and identification, and in turn, require a transformation of parental functions [41]. Perceived parenting is crucial for adolescents’ development and well-being. Indeed, positive and negative parenting perceptions alter not only the relationship with parents but also adolescent development since it can affect emotional adjustment [42], attitudes towards themselves, quality of relationships with peers [43,44], and the internalization and externalization of symptoms [41,45]. Given the important role played by fathers in the family context and in accordance with Steinberg and Silk’s [4] model of parenting, the present study aims to investigate three specific dimensions of parenting processes (closeness, communication, and parent’s peer approval), and to compare any differences in perceived maternal and paternal parenting. Through the dimensions of closeness and communication, it is possible to investigate the affective component of the relationship, while through the parent’s approval of peers, it is possible to investigate the balance between growth and independence [4]. These specific dimensions of parenting were investigated, as they are considered fundamental in adolescence since adolescents seek the presence and affection of their parents while claiming autonomy concomitantly. Therefore, it is of paramount importance to maintain a harmonious bond and cohesion between parents and adolescents at a time when it is not so easy to establish an adequate distance.

#### 1.3.1. Parent–Adolescent Closeness 

Closeness assesses affectionate behaviors and the strength of emotional bonding, which is the ability of parents to connect emotionally and interact with each other in positive and productive ways, such as parental praise, approval, encouragement, physical affection, and displays of love and acceptance [46]. A strong bond between parents and adolescents provides a safer environment, allowing adolescents to explore their growing freedom and continue to be supported through the attachment bond with their parents. Adolescents who have a deep sense of bonding with their parents show better adaptive functioning [47,48,49], better emotion regulation skills, and consequently might exhibit higher levels of flourishing [16].

#### 1.3.2. Parent–Adolescent Communication 

Communication assesses the level of openness and the extent to which adolescents feel free to talk with their mothers and fathers about their problems, personal decisions, or work projects [50]. During the adolescent period, family communication becomes more critical because parents must adapt to the rapid development of adolescents and define boundaries [33]. The quality of communication between parents and adolescents can potentially influence several areas of adolescent development, including flourishing. Family communication problems (e.g., hesitation to reveal personal feelings and avoidance of talking about certain topics) can lead to misunderstandings and conflicts between parents and adolescents, which can reduce flourishing [15].

#### 1.3.3. Parent’s Peer Approval 

A parent’s peer approval assesses whether parents accept the adolescents’ friends or acquaintances. During the adolescent period, teenagers increasingly see friendship as an area over which they should have control and not their parents. Conversely, parents are concerned about their children’s choice of friends as they argue that peer relationships are often linked to other issues, such as the moral sphere [51]. Thus, it would be necessary to strike a balance between the adolescents’ needs and parents’ concerns since parental approval of peers helps adolescents to experience their social world with a greater degree of autonomy, a more positive sense of self, and better adaptation [52]. 

### 1.4. Flourishing, Self-Control, and Perceived Parenting

Because the underlying neurological processes involving the prefrontal cortex are not fully developed in adolescence, self-control is considered a baseline variable because it has an early development that continues throughout adolescence [53]. Several studies demonstrated that self-control is crucial for promoting individual and social well-being. In reality, self-control plays a central role in many aspects of life, including academic success, prosocial behavior, reduced involvement in delinquency, substance use [54,55], and relationship with parents [55,56]. Adolescents with high self-control are more positive about their parents and parent–adolescent relationships [55,56]. In turn, parenting appears to promote well-being through tangible, emotional, informational, and motivational support [57,58,59]. In this regard, Orkibi et al. [12] showed a positive relationship between self-control skills and flourishing, as well as an indirect link via adolescents’ perceived social support from parents and peers. 

This study aims to contribute to the existing literature by proposing specific measures for assessing adolescents’ personal and interpersonal resources, such as self-control, flourishing, and perceived dimensions of maternal and paternal parenting, and to investigate, through a multidimensional approach, adolescents’ perceptions of parenting by considering possible differences between maternal and paternal parenting with respect to the main dimensions of parenting in adolescence (closeness, communication, and peer approval). The overall objective of this study was to investigate how in adolescence, the still-mature ability to exercise self-control promotes well-being directly and indirectly through three specific dimensions of perceived maternal and paternal parenting: communication, closeness, and peer approval by the parent. 

## 2. Materials and Methods

### 2.1. Participants and Procedures

The sample consisted of 335 Italian adolescents aged 14 to 18 (mean age = 16.12, SD = 1.37; 39.4% male), from working and middle-class families. According to data retrieved from the Italian Ministry of Education (www.miur.gov.it, accessed on 3 April 2023), about 95% (*n* = 321) of the participants were Italian nationals and were selected in two secondary schools from urban and suburban school districts in northern Italy. The majority came from middle-class families, with the majority of parents (76%) having a high school diploma or university degree. All participants indicated that they were not hospitalized due to psychiatric symptoms in the past two years. Few participants (<5% of the total sample) reported previous counseling or psychological interventions in the past two years for mild problems, such as school problems and/or short-term emotional disturbances. No participant was excluded due to their psychiatric history. Participation was entirely voluntary with informed parental consent required before students could participate. The administration was conducted in compliance with the ethical standards for research outlined in the Ethical Principles of Psychologists and the Code of Conduct [51]. The power analysis revealed that 89 subjects were required for adequate study power. This study was approved by the University’s Ethics Committee (ID.1523/15).

### 2.2. Measures

The Flourishing Scale (FS) [6,57] is a brief 8-item measure of the respondent’s self-perceived success in important areas such as relationships, self-esteem, purpose, and optimism. Respondents are asked to rate items on a 7-point Likert scale, ranging from 1 = strongly disagree to 7 = strongly agree. High scores signify that respondents view themselves in positive terms across diverse domains that are widely believed to be important. Sample items are: “I lead a purposeful and meaningful life”; “Social relationships are supportive and rewarding”. The measure has good psychometric properties and is strongly associated with other psychological well-being scales [58]. In the present study, composite reliability was 0.75.

Adolescent Family Process (AFP) [50,59] investigates specific dimensions of maternal and paternal parenting as assessed by adolescents. AFP consists of 25 maternal and 25 paternal items across six subscales: closeness, support, communication, monitoring, peer approval, and conflict. Three subscales were used in this study: closeness, communication, and peer approval. The closeness subscale consists of 6 items and evaluates affectionate behaviors and the strength of the emotional attachment (e.g., “My mother gives me the right amount of affection”; “My father trusts me”). The communication subscale consists of 5 items that measure the level of openness and, in particular, the extent to which adolescents feel free to approach their mothers and fathers about their problems, personal decisions, or job plans (e.g., “How often do you talk to your mother about other things that are important to you?” and “How often do you talk to your father about major personal decisions?”). The peer approval subscale consists of 3 items and measures whether parents accept adolescents’ friends or dates (e.g., “How often does your mother approve of your friends?” and “How often does your father like when you go out with your friends?”). These dimensions, separately for father and mother, are rated on a five-point Likert scale, ranging from 1 (strongly disagree or never) to 5 (strongly agree or very often). In the present study, composite reliability for maternal closeness, communication, and peer approval were 0.72, 0.73, and 0.73, respectively. Composite reliability for paternal closeness, communication, and peer approval were 0.72, 0.72, and 0.73, respectively.

The Brief Self-Control Scale (BSCS) [10,49] assess participants’ ability to control their impulses, emotions, and thoughts, interrupt undesired behavioral tendencies, and refrain from acting on them. The scale includes 13 items rated on a 5-point Likert scale (from 1 = “not like me at all” to 5 = “very much like me”), with higher scores indicating greater self-control. Sample items are: “I am good at resisting temptation”; “Sometimes I can’t stop myself from doing something, even if I know it is wrong”. In the present study, composite reliability was 0.77.

### 2.3. Data Analysis

All statistical analyses were performed using the statistical package for the social sciences (IBM SPSS version 22). A paired *t*-test with bootstrapped confidence intervals based on 5000 replications was used to analyze the differences between maternal and paternal dimensions of parenting. Cohen’s d [60] was used to examine whether the differences were substantial. Pearson correlation analyses were conducted to explore the associative pattern between self-control, flourishing, maternal and paternal perceptions of closeness, communication, parent’s peer approval, and demographic variables (age and sex). Process analysis was performed using the SPSS macro (PROCESS) [61]. We did not use structural equation modeling because this study aimed to evaluate observed variables and not latent variables. Hayes et al. [62] suggests using PROCESS for models that rely entirely on observed variables because the use of SEM may be susceptible to measurement error. Before analyzing the mediating effects of maternal and paternal closeness, communication, and peer approval in the association between self-control and flourishing, multicollinearity between independent variables was tested. It was determined that there is no multicollinearity between independent variables based on tolerance ≥ 0.1 (0.43–0.84), and variance inflation factor (VIF) < 10 (1.19–2.39). Furthermore, the Durbin–Watson value of 2.12 indicates the absence of autocorrelation. However, the requirements of normality and homoscedasticity were not met; this could be due to the sample size. Hayes [63] considers these two assumptions to be of minor importance and suggests that heteroscedasticity could be determined by the sample size and that only the most serious violations of the normality assumption substantially affect the validity of statistical inferences from a regression analysis. The PROCESS 4.0 model was evaluated three times, considering self-control as the independent variable, flourishing as the dependent variable, and maternal and paternal closeness, communication, and parent’s peer approval as mediators, respectively. Through these models, it was possible to investigate how direct and indirect effects of self-control, mediated by perceived parenting dimensions, were associated with adolescents’ level of flourishing. First, the total effect of self-control on flourishing was calculated without including the mediator of perceived parenting. The aim was then to include mediators to test the direct and indirect effects of parental perception on flourishing. This made it possible to observe the variation in total effects and the influence of mediators (i.e., the mediated effect). To test the mediated effects, the bootstrap method was applied. Specifically, 5000 bootstrap samples were drowned from the complete data and a 95% confidence interval was used to determine the significance of the mediated effects. Significant effects would were identified if the confidence interval excluded 0. It should be noted that PROCESS’ mediational models adopt the ordinary least square (OLS) regression method to estimate models’ effects [64], meaning that associations between variables are assessed while controlling for the influence of all others included in the model. Through the OLS regression method, it is thus possible to control for the variables’ shared variance, thereby justifying performing mediational analysis on cross-sectional data in which no causal relations can be implied. Accordingly, terms such as “effect”, “influence”, or “mediate” will be used in line with the model performed, yet not to suggest causality.

## 3. Results

### 3.1. Comparing Maternal and Paternal Dimensions of Perceived Parenting

To compare the perception of maternal and paternal closeness, communication, and parent’s peer approval, paired sample t-tests was used, as shown in Table 1. Adolescents perceived significantly more closeness, communication, and parent’s peer approval in association with mothers than fathers. However, Cohen’s d was lower than 0.50, showing that the difference was not substantial (Table 1).

### 3.2. Correlation between the Main Variables in This Study

Descriptive statistics and the Pearson correlations among self-control, maternal and paternal closeness, communication, parent’s peer approval, flourishing, and demographic variables are shown in Table 2. Self-control was significantly correlated in the expected direction with flourishing and maternal and paternal parenting dimensions. The latter correlates in the expected direction with flourishing (Table 2). Sex was significantly correlated with the three dimensions of maternal parenting with paternal peer approval and self-control, while age correlates with maternal peer approval and paternal closeness.

### 3.3. The Mediational Model

#### 3.3.1. Self-Control, Maternal and Paternal Closeness, and Flourishing 

**Figure 1:**
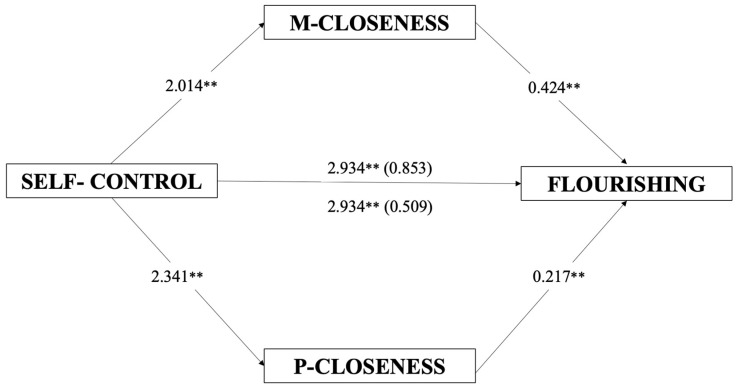
Mediation model. Direct and indirect effects of self-control on flourishing, mediated by maternal and paternal closeness. Note. Slope coefficients are unstandardized; numbers in parentheses represent a significant indirect effect mediated by the presence of maternal and paternal closeness (5000 bootstrap samples, 95% confidence interval). M = maternal; P = paternal. ** *p* < 0.01.

The mediational model investigated the direct and indirect effects of self-control on flourishing, mediated by maternal and paternal closeness (see Table 3 and Figure 1). The full model was significant (*F*(5, 329) = 22.36 *p* < 0.001) and accounted for about 25% of the explained variability in the flourishing (*R*^2^ = 0.254). Self-control showed a significant positive direct effect (*B* = 2.934, *t* = 5.04, *p* < 0.001) on flourishing, and a significantly positive effect on maternal and paternal closeness (*B* = 2.014, *t* = 5.48, *p* < 0.001; *B* = 2.341, *t* = 4.99, and *p* < 0.001). Maternal and paternal closeness showed a statistically positive effect on flourishing (*B* = 0.424, *t* = 4.97, *p* < 0.001; *B* = 0.217, *t* = 3.25, and *p* < 0.01). Self-control showed an indirect effect on flourishing as mediated through maternal and paternal closeness (*B* = 0.853, CI = 0.342, 1.539; *B* = 0.509, and CI = 0.175, 0.941). 

#### 3.3.2. Self-Control, Maternal and Paternal Communication, and Flourishing 

**Figure 2:**
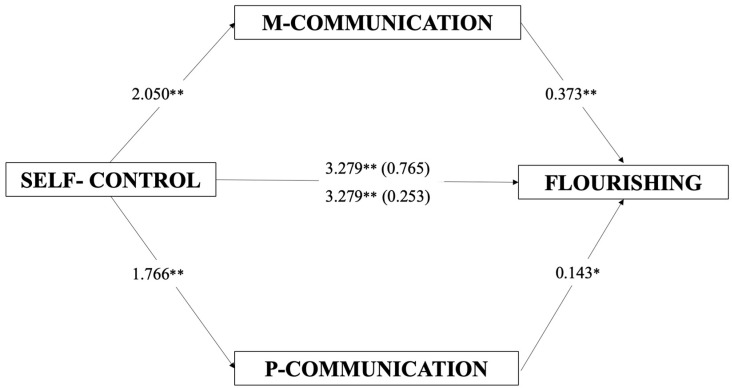
Mediation model. Direct and indirect effects of self-control on flourishing, mediated by maternal and paternal communication. Note. Slope coefficients are unstandardized; numbers in parentheses represent a significant indirect effect mediated by the presence of maternal and paternal communication (5000 bootstrap samples, 95% confidence interval). M = maternal; P = paternal. ** *p* < 0.01, * *p* < 0.05.

The mediational model investigated the direct and indirect effects of self-control on flourishing, mediated by maternal and paternal communication (see Table 4 and Figure 2). The full model was significant (*F*(5, 329) = 20.04 *p* < 0.001) and accounted for about 23% of the explained variability in flourishing (*R*^2^ = 0.233). Self-control showed a significant positive direct effect (*B* = 3.279, *t* = 5.67, *p* < 0.001) on flourishing, and a significantly positive effect on maternal and paternal communication (*B* = 2.050, *t* = 4.98, *p* < 0.001; *B* = 1.766, and *t* = 3.76, *p* < 0.001). Maternal and paternal communication showed a significantly positive effect on flourishing (*B* = 0.373, *t* = 4.70, *p* < 0.001; *B* = 0.143, and *t* = 2.06, *p* < 0.05). Self-control showed an indirect effect on flourishing as mediated through maternal and paternal communication (*B* = 0.765, CI = 0.302, 1.344; *B* = 0.253, and CI = 0.016, 0.633)

#### 3.3.3. Self-Control, Maternal and Paternal Peer Approval, and Flourishing 

**Figure 3:**
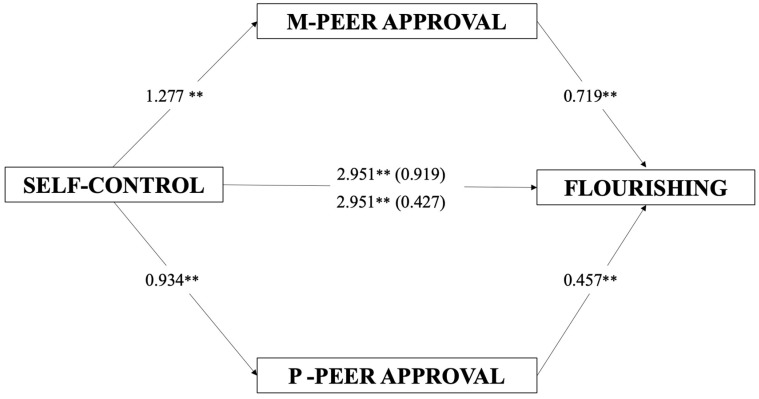
Mediation model. Direct and indirect effects of self-control on flourishing, mediated by maternal and paternal peer approval. Note. Slope coefficients are unstandardized; numbers in parentheses represent a significant indirect effect mediated by the presence of maternal and paternal peer approval (5000 bootstrap samples, 95% confidence interval; M = maternal; P = paternal. ** *p* < 0.01.

The mediational model investigated the direct and indirect effects of self-control on flourishing, mediated by maternal and paternal peer approval (see Table 5 and Figure 3). The full model was significant (*F*(5, 329) = 23.93 *p* < 0.001) and accounted for about 26% of the explained variability in flourishing (*R*^2^ = 0.267). Self-control showed a significant positive direct effect (*B* = 2.951, *t* = 5.14, *p* < 0.001) on flourishing, and a significantly positive effect on maternal and paternal peer approval (*B* = 1.277, *t* = 6.20, *p* < 0.001; *B* = 0.934, and *t* = 3.82, *p* < 0.001). Maternal and paternal peer approval showed a statistically positive effect on flourishing (*B* = 0.719, *t* = 4.48, *p* < 0.001; *B* = 0.457, and *t* = 3.39, *p* < 0.001). Self-control showed an indirect effect on flourishing as mediated through maternal and paternal peer approval (*B* = 0.919, CI = 0.359, 1.588; *B* = 0.427, and CI = 0.152, 0.799).

## 4. Discussion

Flourishing is an area of research that is gaining much interest. The United Nations Convention on the Rights of the Child (UNCRC) [65] emphasized the importance of promoting flourishing in adolescents, as there are several significant changes at this stage of life that could compromise well-being and development [1]. Flourishing in adulthood is associated with prosocial behavior [66], the implementation of a healthy lifestyle [67], improved school performance [68], enhanced creativity [69], gratitude, optimism, self-esteem, and happiness [70]. Furthermore, flourishing was found to reduce the risk of mood disorders such as anxiety and depression [25,71]. These findings highlight the need to foster the development of flourishing in adolescence as a public health issue [72]. In this regard, some studies [12,73] defined the relationship with parents as one of the main protective factors of flourishing in adolescence, which is correlated with self-control [12].

In this regard, this study set out to investigate the protective factors involved in flourishing during adolescence [74]. The results of this study reveal a significant positive correlation between self-control, the three examined dimensions of maternal and paternal parenting (closeness, communication, and parental peer approval), and the flourishing of adolescents, as controlling thoughts, emotions, and impulses enabled them to follow social norms and personal values, and thus promoted interpersonal relationships [14] as well as flourishing [12,13]. Instead, correlations with age and sex had small effect sizes and hence will not be subject for interpretation. Parent–adolescent relationships characterized by closeness, communication, and parent’s peer approval increased adolescents’ levels of flourishing [12,13,15,16,17]. Furthermore, the model examined in the present study showed that adolescents’ self-control skills are correlated directly and indirectly with flourishing across three dimensions of perceived maternal and paternal parenting. Self-control, in fact, exercising control over thoughts, emotions, and behavior [15,16,17], can help adolescents achieve a higher level of flourishing. Furthermore, individuals with high self-control skills may feel more comfortable seeking parental closeness and support because they are less concerned about relinquishing personal control, thereby increasing levels of flourishing [75]. 

This study expanded the literature on flourishing by identifying self-control, closeness, communication, and maternal and paternal peer approval among the factors that favor the development of flourishing in adolescence. Furthermore, the present study, unlike the numerous studies in the literature, assessed flourishing in adolescence through a specific measure. Furthermore, the study investigated three aspects of parenting in mothers and fathers individually. Separate attention was given to mothers and fathers, allowing for more precise information about the significance of self-control and relationships with parents as protective factors for flourishing in adolescence. There were not substantial differences between levels of maternal and paternal support, closeness, and parent’s peer approval as perceived by adolescents. However, in mediation models, the mother’s influence was found to be greater than that of the father. As for support and communication, these results seem to confirm previous literature on relationships with mothers versus fathers in adolescence [76,77]. The mother–adolescent relationship was suggested to be characterized by greater closeness, more open communication, and peer approval on the part of the parent, as mothers compared to fathers, especially during childhood, spend more time with their children, are more involved in their lives, and hold most of the responsibility for tasks such as daily care [76]. The constant presence of the mother during childhood seems to partly determine a more intimate, affective, and cohesive relationship with the children during adolescence [77]. However, both parents seemed to have a greater influence on parents’ peer approval than communication and closeness. This latter result may be related to the specific developmental period and the crucial role played by peers and parents in facilitating the separation–individuation process in the adolescent period [78].

### Limitations

This study has some limitations. First, the sample was community-based, but only a few schools were invited to participate, which makes the sample unrepresentative and limits the generalizability of this research. All variables were evaluated with self-reports; therefore, the variance of the shared method possibly played a role in some of the associations between the variables. In the relationship analysis between the three constructs (self-control, perceived parenting, and flourishing) only two socio-demographic variables (age and sex) were considered, therefore, future studies could also investigate other variables that might be associated with flourishing, such as religiosity and family characteristics. Furthermore, a cross-sectional design was used, which does not allow a cause-and-effect relationship to be deduced. It would also be interesting to evaluate flourishing longitudinally and investigate all dimensions of parenting, focusing more on the dimension of conflictual, which was not considered in this study, but the importance of which is recognized as relationships in this period of life often tend to be turbulent and conflictual [79]. 

Despite these limitations, it is believed that the study provided interesting preliminary results, which could lay the foundation for future research on flourishing in adolescence by considering two constructs central to flourishing [80]: self-control and parenting.

## 5. Conclusions

The present study, through the identification of protective factors of flourishing in adolescence, namely self-control and relationships with one’s parents characterized by proximity, communication, and parent’s peer approval, made an important contribution to the literature on flourishing. These findings, in fact, highlight the need to promote self-control and parent–child relationships during adolescence, with the aim of increasing flourishing levels. Therefore, this study showed the importance of assessing the role of parenting in mothers and fathers and makes a specific contribution to the existing literature on flourishing, which is currently lacking [25], especially in adolescence. At last, the current study demonstrates the importance of flourishing in adolescence and highlights the need for greater public health interest in promoting public projects and services that foster and increase adolescent flourishing.

## Figures and Tables

**Table 1:** The *t*-test for paired samples among the perception of maternal and paternal closeness, communication, and parent’s peer approval.

	Maternal		Paternal		*t*	CI (95%)	Cohen’s *d*
	M	SD	M	SD			
Closeness	23.89	4.17	21.35	5.27	−8.33 **	[0.200, 0.435]	0.45
Communication	16.93	4.73	14.27	5.12	−8.67 **	[0.233, 0.468]	0.47
Peer approval	12.10	2.35	11.60	2.68	−3.41 **	[0.327, 0.552]	0.19

Note. CI = confidence interval; ** *p* < 0.01; and * *p* < 0.05.

**Table 2:** Mean values, standard deviation, and Pearson correlations among self-control, maternal and paternal closeness, communication, peer approval, flourishing, and demographic variables.

	M	SD	1	2	3	4	5	6	7	8	9	10
1. Self-control	3.31	0.59										
2. M-Closeness	23.89	4.17	0.31 **									
3. P-Closeness	21.35	5.27	0.25 **	0.32 **								
4. M-Communication	16.93	4.73	0.29 **	0.63 **	0.17 **							
5.P-Communication	14.27	5.12	0.20 **	0.20 **	0.66 **	0.35 **						
6. M-Peer approval	12.10	2.35	0.33 **	0.53 **	0.27 **	0.46 **	0.22 **					
7. P-Peer approval	11.60	2.68	0.18 **	0.21 **	0.55 **	0.11 **	0.46 **	0.44 **				
8. Flourishing	42.16	6.71	0.36 **	0.37 **	0.31 **	0.34 **	0.26 **	0.40 **	0.35 **			
9. Age	16.12	1.37	−0.01	−0.03	−0.20 **	0.10	−0.10	0.12 *	0.01	0.02		
10. Sex			0.14 **	0.20 **	−0.03	0.26 **	−0.03	0.12 *	0.21 **	−0.06		

Note. M = maternal; P = paternal. ** *p* < 0.01; and * *p* < 0.05.

**Table 3:** Unstandardized coefficients of the mediation model of self-control, maternal and paternal closeness, and flourishing.

Paths		Direct Effects				Indirect Effects	
	*B*	SE	*t*	*p*	*B*	SE	CI (95%)
Total sample (*N* = 335)							
Self-control → Flourishing	2.934	0.58	5.04	<0.001			
Self-control → M-Closeness	2.014	0.37	5.48	<0.001			
Self-control → P-Closeness	2.341	0.47	4.99	<0.001			
M-Closeness → Flourishing	0.424	0.08	4.97	<0.001			
P-Closeness → Flourishing	0.217	0.07	3.25	<0.001			
Self-control → M-Closeness → Flourishing					0.853	0.30	[0.342, 1.539]
Self-control → P-Closeness → Flourishing					0.509	0.20	[0.175, 0.941]

Note. CI = confidence interval; M = maternal; and P = paternal.

**Table 4:** Unstandardized coefficients of the mediation model of self-control, maternal and paternal communication, and flourishing.

Paths		Direct Effects				Indirect Effects	
	*B*	SE	*t*	*p*	*B*	SE	CI (95%)
Total sample (*N =* 335)							
Self-control → Flourishing	3.279	0.58	5.67	<0.001			
Self-control → M-Communication	2.050	0.41	4.98	<0.001			
Self-control → P-Communication	1.766	0.47	3.76	<0.001			
M-Communication → Flourishing	0.373	0.08	4.70	<0.001			
P-Communication → Flourishing	0.143	0.07	2.06	<0.05			
Self-control → M-Communication → Flourishing					0.765	0.26	[0.302, 1.344]
Self-control → P-Communication → Flourishing					0.253	0.16	[0.016, 0.633]

Note. CI = confidence interval; M = maternal; and P = paternal.

**Table 5:** Unstandardized coefficients of the mediation model of paternal dimensions self-control, maternal and paternal peer approval, and flourishing.

Paths		Direct Effects				Indirect Effects	
	*B*	SE	*t*	*p*	*B*	SE	CI (95%)
Total sample (*N =* 335)							
Self-control → Flourishing	2.951	0.57	5.14	<0.001			
Self-control → M-Peer approval	1.277	0.20	6.20	<0.001			
Self-control → P-Peer approval	0.934	0.24	3.82	<0.001			
M-Peer approval → Flourishing	0.719	0.16	4.48	<0.001			
P-Peer approval → Flourishing	0.457	0.13	3.39	<0.001			
Self-control → M-Peer approval → Flourishing					0.919	0.31	[0.359, 1.588]
Self-control → P-Peer approval → Flourishing					0.427	0.17	[0.152, 0.799]

Note. CI = confidence interval; M = maternal; and P = paternal.

## Data Availability

The data presented in this study are available on request from the corresponding author.
